# Case of Walter Palmer

**Published:** 1856-03

**Authors:** 


*Case of Walter Palmer.*—We continue, according to promise, our account of the Rugeley mysteries, in order, as we stated, that our readers may trace the successive points that are brought out in evidence, and may possess, for future reference, a record of transactions which must of necessity be constantly referred to hereafter in the annals of Medical Jurisprudence.

The case of Walter Palmer is this: It is suspected, or alleged, that his death was compassed or promoted by his brother William; and that the motive for this supposed crime was the desire to obtain a large sum of money, for which Walter Palmer's life was insured.

Our position as Editor of a leading Medical Periodical, enables us to obtain, from many sources, authentic accounts of the evidence which has been, as well as of that which will be, brought forward on various sides of the case; but we deem it our duty not to divulge any matters of criminatory importance before they are delivered as testimony in open court. We shall take the alleged facts as to Walter Palmer's death first, reserving the important particulars as to the insurances, in order to see if they are made public before we go to press.

Walter Palmer was aged 32, said to be of easy temper, unfortunate in business, addicted to drink, and, on this account, living away from, but seemingly not on bad terms with his wife, a lady whose misfortunes and straightforward character entitle her to all due sympathy and respect. The account we have of him is, that in 1852, '53, and '54, he had been laboring under great enlargement of the liver, dropsy, vomiting of blood, and pleuro-pneumonia—the disease of drunkards *par excellence;* moreover, that he had suffered from two distinct attacks of *delirium tremens;* and yet, that his life was insured for £13,000 at a heavy premium by the Prince of Wales Office, in January, 1855. During the spring of 1855, Walter Palmer was seen by several Medical gentlemen, and by other witnesses; and from their united testimony, we gather that his health was then not so bad, in outward appearance, as might be imagined from the effects of previous intemperance, and that his habits were temperate for him, although far from temperate in the abstract. For example, when on what one witness called the "sober tack," his allowance was half or three quarters of a bottle of gin daily; and yet, during the months between January and July, 1855, it must be remembered that repeated attempts were made to increase the sums for which his life was insured.

In the month of July, we get a more explicit and vivid picture of him; for, on the 7th of that month, Mr. Henry Day, surgeon of Stafford, was called in to see Walter Palmer by William Palmer. The latter described his brother as being exceedingly ill through intemperance; that this was a source of great anxiety to the family, and that Mr. Day would earn their everlasting gratitude if he could induce him to reform. Mr. Day appears to have visited his patient, to have discovered chronic inflammation of the lungs, and to have prescribed medicines well suited to the patient's malady and habits. The patient seems at this time to have been in a state of recklessness; "not clean" in his person; and refusing to take the serious advice which Mr. Day very properly gave him. On the 10th of August, Mr. Day's opinion of his health may be gathered from the fact, that he told him that "he would some day shut up like a knife,"—a prophecy destined to speedy fulfilment;—and as to his mental condition, Mr. Day affirms that he never could tell whether he was speaking the truth or not.

Hence it is quite vain to attempt to interpret a suspicious observation made by Walter Palmer to Mr. Day, about the 10th or 12th of August, to the effect that some pills which he had taken were "twisters." Now, Mr. Day had given no pills; and it has been surmised that William Palmer gave his brother, surreptitiously, some pills intending to do him no good, pretending that they came from Mr. Day. But the mental condition described by Mr. Day, renders it quite impossible to say, first, whether Walter Palmer meant what he said, or said what he meant; secondly, if so, whether it was or was not true. Very little can be built upon this.

Mr. Day's attendance ceased, for a time, on the 31st of July. After this, it appears that Walter Palmer went to Liverpool with his wife, and remained four days, during which time she describes him as *perfectly sober*. But, on his return to Stafford, on the 8th or 9th, and from that time to his decease, on the 16th, there is but one account from all who who saw him, and that he was night and day in a state of constant brutalizing intoxication; day after day poisoned with immense quantities of gin; vomiting every morning; eating "an ounce out of a mutton chop" for his dinner, going to bed with a gin-bottle at his elbow, and not sober when he woke in the morning. On the 10th of August, Mr. Day again saw him, and repeated his visit at intervals up to the 16th, on which morning the last act of this tragedy took place. Mr. Day being called to him on that morning, found him *in articulo mortis*—countenance bluish, convulsive twitchings of left side of face; pulse gone; a little yellow liquid running out of the corner of the mouth, which smelt like brandy-and-water;—and there was an end of Walter Palmer!

Thomas Walkeden, a man with whom Walter Palmer lodged, and whose account of the habits of the deceased, and of his own occupation in supplying him with drink, is truly horrible, describes, on the morning of the 16th, "a sudden change in his face, which became black for about half-a-minute; his eyes flushed in a peculiar way, and he had a difficulty in breathing." This change had been noticed by Walkeden previously, when Walter had been drinking hard, and it is not a bad description of threatening coma.

From the facts foregoing, it was natural enough that Mr. Day should give, as he did, a certificate that death was the result of apoplexy, arising from visceral disease; and probably most of our readers will agree with Mr. Robert Hughes, one of the Medical witnesses, that the patient was sufficiently *poisoned* with ardent spirits to put an end to his life.

But there remains in the background a series of unexplained circumstances as to the conduct of William Palmer. How any man, within six months, could repeatedly concur in proposing such a life for insurance, we know not, nor does it much matter. Graver matter remains. On Tuesday, August 14, two days before his brother died, William Palmer is sworn to have bought prussic acid at Wolverhampton. On the same day he gave two bottles to the "Boots" at the Grand Junction Hotel at Stafford, to be taken care of. On August 15, he asked for the bottles, took them into the stable, was seen by the "Boots" and the Landlord to pour a liquid out of one of them into another bottle, and add to the contents of one a colorless liquid, like water. He said that his brother was very ill, that he was going to take him "something stimulating;" that Mr. Day did not know his brother's constitution so well as he did. He was with his brother on the morning of the 16th; and the "fit," which carried him off in half-an-hour, came on while William Palmer was there.

Such an act was unprofessional towards Mr. Day, and suspicious in itself. The accounts as to the number and date of his visits to his brother, during the last few days of his life; his procuring brandy for a man already dying of gin; his not sending for his brother's wife—all these points want clearing up; and no doubt the kind of doubts hanging over the case induced the jury, notwithstanding the evidence that the deceased had taken spirits enough to poison twenty men, to return a verdict to the effect that Prussic Acid had been administered by William Palmer.

Be it remarked, that all that is substantiated by evidence is, that the circumstances of Walter Palmer's death *are not incompatible* with the hypothesis that prussic acid was administered.

Of course the verdict of the coroner's jury, like that of a grand jury, must be taken to intimate no more than that there is *prima facie* ground for further proceedings, in the course of which we are bound charitably to hope that William Palmer will be able to clear himself from the horrible suspicion which now rests upon him.

Here we must pause, with a passing allusion to the "Boots," who declares that some brandy, which William Palmer gave him after his brother's death, made him sick. We know for a fact, and ludicrous enough it is, that almost everybody, or rather every blackleg, who has eaten or drunk at William Palmer's table, now pretends to recollect that he tasted something odd in the brandy-and-water, or that he felt sick in the night!—*Medical Times and Gazette.*

